# Exonic deletions in *GALC* are frequent in Japanese globoid-cell leukodystrophy patients

**DOI:** 10.1038/s41439-018-0027-5

**Published:** 2018-10-05

**Authors:** Kaori Irahara-Miyana, Takashi Enokizono, Keiichi Ozono, Norio Sakai

**Affiliations:** 1 Department of Pediatrics, Osaka University Graduate School of Medicine, Osaka, Japan; 2 Department of Pediatrics, University of Tsukuba Hospital, Ibaraki, Japan; 3 Child Healthcare and Genetic Science, Department of Health Science, Osaka University Graduate School of Medicine, Osaka, Japan

## Abstract

Globoid-cell leukodystrophy is an autosomal-recessive lysosomal storage disorder. Single-base substitutions and small indel mutations in the *GALC* gene are common in Japanese patients. In this study, we identified three novel deletions, in exons 1, 8, and 11–12, in three patients using Multiplex Ligation-dependent Probe Amplification. We suggest that some patients in whom no or only a single pathogenic mutation is detected by Sanger sequencing may have exon deletions.

Globoid-cell leukodystrophy (GLD; MIM #245200), also known as Krabbe disease, is an autosomal-recessive lysosomal storage disorder resulting from a deficiency of galactocerebrosidase (GALC) activity^1^ with the mutation of the responsible gene of *GALC*. The *GALC* gene is located at 14q13, and comprises an open reading frame of 2007 base pairs, composed from 17 exons^2^. Clinically, a deficiency in GALC results in widespread central and peripheral nervous system demyelination and leads to various symptoms, including developmental delay, visual impairment, spastic diplegia, and cognitive disorders. The spectrum of phenotypic manifestations is classified into infantile, late infantile, juvenile, and adult forms according to the age of onset. To date, more than 140 mutations in the *GALC* gene have been reported. A 30-kb deletion is common in patients from Europe^3^, whereas seven common mutations (NM_000153.3:c.683_692delinsC (p.Met227_Tyr231delinsThr), c.2002A > C (p.Thr668Pro), c.658C > T (p.Arg220*), c.952C > G (p.Pro318Ala), c.1901T > C (p.Leu634Ser), c.246A > G (p.Ile82Met) + c.913A > G (p.Ile305Val), and c.857G > A (p.Gly286Asp)) account for 58% of mutant alleles of patients with GLD in Japan^4^. However, there are several patients in whom either only one or no *GALC* mutation has been identified by Sanger sequencing of each exon. Therefore, we investigated deletion/duplications of the 17 exons of *GALC* using the Multiplex Ligation-Dependent Probe Amplification (MLPA) assay, which is a quantitative multiplex polymerase chain reaction (PCR) approach, to quantify relative copy numbers in each exon of the *GALC* gene of five patients from whom DNA was available. This is the first report to our knowledge that analysed Japanese GLD patients with the MLPA method.

The biochemical diagnosis of GLD is based on low GALC enzymatic activity. Enzyme activity was measured using 6-hexadecanoylamino-4-methylumbelliferyl-β-d-galactopyranoside (Slater & Frit Ltd., Norwich, UK) as an artificial fluorescence substrate following a reported protocol^5^. Briefly, patient lymphocytes were sonicated and incubated with the substrate in citrate-phosphate buffer (pH 4.2) at 37 °C for 4 h, and fluorescence (excitation at 385 nm/emission at 450 nm) was measured using a microplate reader. Enzyme activity was calculated in nmol/h/mg protein.

After informed consent was obtained, genomic DNA was prepared using standard methods from patient peripheral blood lymphocytes or cultured skin fibroblasts. PCR reactions and Sanger sequencing were performed as previously described^6^. The primers for exon 1 were revised from a previous paper, and the sense primer (5′-cctcctgcccgtatctatcgtg-3′) and antisense primer (5′-tgactggcaccctaggggaat-3′) were used. All exons were analysed by the MLPA method using the SALSA MLPA kit P446 for *GALC* (MRC-Holland, Amsterdam, the Netherlands) according to the manufacturer’s protocol. After the MLPA assay, Sanger sequencing was performed using a sense primer (5′-cttattaacttttctctcagtccctcactc-3′) and an antisense primer (5′-actttactctctgcacctatactctctgg-3′), and the patient was identified as having a heterozygous deletion of exon 1.

The genome of 31 patients who were diagnosed as having GLD based on deficiency of GALC enzyme activity were subjected to Sanger sequencing from 2009 to 2016 using the Sanger method, and in 11 of them, only one or no mutation was identified. Of these 11 patients, five whose DNA samples were available were subjected to the MLPA assay. For the five patients with GLD, the clinical phenotypes were infantile type (*n* = 3), late infantile type (*n* = 1), and adult type (*n* = 1) (Table 1). In three patients, one previously reported pathogenic mutation was identified. In two patients, no mutation was identified (Table 1). MLPA showed a decrease in the signal intensities for exons in *GALC* in three patients: NM_000153.3:c.(?_-29)_(195 + 1_196-1)del (exon 1) for patient 1, NM_000153.3:c.(752 + 1_753-1)_(908 + 1_909-1)del (exon 8) for patient 2, and NM_000153.3:c.(1033 + 1_1032-1)_(1251 + 1_1252-1)del (exon 11 and 12) for patient 3 (Fig. 1a). No duplications or deletions were detected in patients 4 and 5. To identify the deletion region in patient 1, we performed Sanger sequencing using another primer set covering the region of exon 1 (Fig. 1b) and the electrophoretogram showed two bands (Fig. 1c). We performed Sanger sequencing of each band and identified a 27 (−32 to −6) nucleotide deletion upstream of the initiation codon (NG_011853.2:c.5368_5394del) in the lower band of patient 1 (Fig. 1d), and no mutation was identified within exon 1. The same deletion was seen in the father.

**Table 1:** GALC enzyme activities in patient lymphocytes, phenotype, genetic analysis

Patient number	GALC enzyme activities (nmol/h/mg protein) (Normal 0.75 ± 0.27)	Phenotype	Genotype	
			Sanger sequence	MLPA
1	0.03	Infantile	[N.D]; [N.D]	c.(?_-29)_(195 + 1_196-1)del
2	0.0	Infantile	c.658 C > T (p.Arg220*); [N.D.]	c.(752 + 1_753-1)_(908 + 1_909-1)del
3	0.28	Infantile	c.175 G > C (p. Gly59Arg); [N.D.]	c.(1033 + 1_1032-1)_(1251 + 1_1252-1)del
4	0.1	Late Infantile	c.1901T > C (p.Leu634Ser); [N.D.]	N.D.
5	0.2	Adult	[N.D.]; [N.D.]	N.D.

*N.D*. not detected

**Fig. 1:**
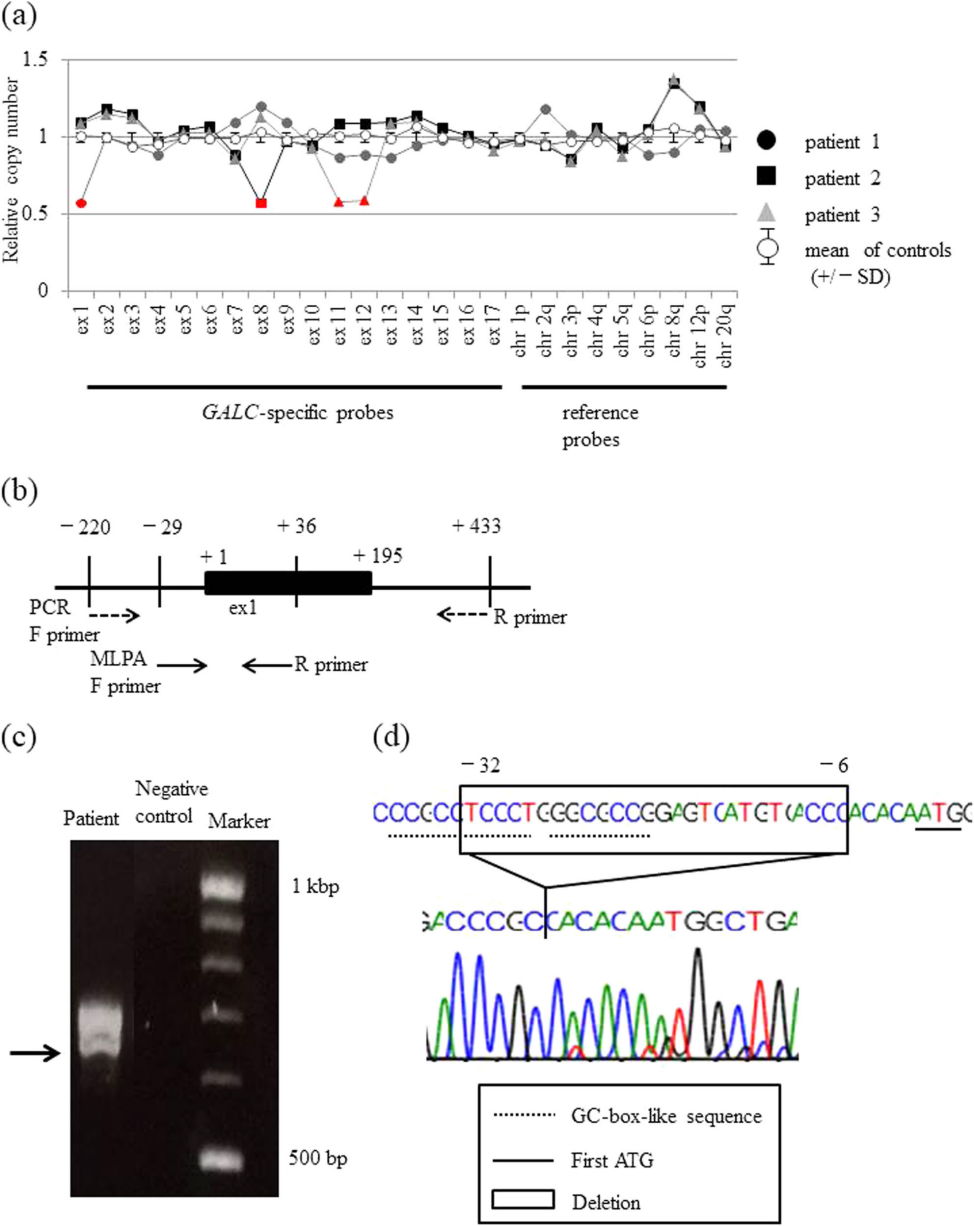
Detection of *GALC* exon 1–14 deletions/duplications by MLPA in GLD patients and a normal control, and a detailed analysis of the *GALC* gene deletion region in patient 1. **a** The panels show the results of the MLPA analyses. The deletions are shown in red. Deletions are detected as a 0.5-fold decrease in the peak height compared with the normal control. **b** The location of each primer used in the MLPA (solid arrow) and PCR (dashed arrow) analyses of exon 1. The analysis region in the PCR assay covers the region of the MLPA analysis. **c** Electrophoresis of *GALC* exon 1 and the region upstream of exon 1. The arrow indicates the smaller band corresponding to the mutant allele. **d** The nucleotide sequence of the lower band in **c**. The original sequence is shown at the top, and the sequence of the lower band in **c** is shown underneath. The region deleted in patient 1 is shown in the box

Common mutations detected in Japanese GLD patients are point mutations and small insertions/deletions; however, the present report suggests that comparatively wide span deletions, including one or two exons, may exist among patients in which pathogenic mutations cannot be identified by Sanger sequencing for each exon. The genotype–phenotype correlation between the exon deletion and phenotype is unknown^7^; however, in the patients in the present study, exon deletions were identified in patients with the infantile form of the disease, and therefore, such deletions may be associated with disease severity. On the other hand, although the MLPA assay can determine gross deletions and duplications, the primers contained in the MLPA kit do not cover each exon fully. Thus, the results of deletions or duplications obtained using the MLPA kit may be different from the actual deletions or duplications. Therefore, it is necessary to perform detailed analyses of the region of deletion/duplication to determine the association between the genetic alteration and its pathogenicity.

In the present report, MLPA suggested that exon 1 was deleted in patient 1, but a detailed investigation demonstrated that the deletion is located in the 5′-untranslated region upstream of the first ATG. This deletion is unreported; however, it includes a highly conserved GC-box-like sequence near the promoter region and a transcription initiation start site^8^, and thus it may have a pathogenic effect on transcription of the *GALC* gene.

One limitation of this study is that we did not identify the region of deletion of patients 2 and 3 because the deletion span was presumed to be longer than several kilobases in patient 2, and the quality of the DNA of patient 3 was poor. Therefore, further investigation is required to confirm the precise deletion region of these patients. This report is the first to our knowledge to analyse Japanese patients with GLD using the MLPA method, and we found a gross deletion including one to two exons in three alleles of five patients in whom two mutations in two alleles could not be detected by Sanger sequencing. These results suggest that genetic analysis using the MLPA method may be useful for patients in whom two mutations are not detected by Sanger sequencing.

## Data Availability

The relevant data from this Data Report are hosted at the Human Genome Variation Database at 10.6084/m9.figshare.hgv.2390 10.6084/m9.figshare.hgv.2393 10.6084/m9.figshare.hgv.2396
